# MINOCA Associated with a Myocardial Bridge: Pathogenesis, Diagnosis and Treatment

**DOI:** 10.3390/jcm12113799

**Published:** 2023-05-31

**Authors:** Riccardo Rinaldi, Giuseppe Princi, Giulia La Vecchia, Alice Bonanni, Giovanni Alfonso Chiariello, Alessandro Candreva, Felice Gragnano, Paolo Calabrò, Filippo Crea, Rocco A. Montone

**Affiliations:** 1 Department of Cardiovascular and Pulmonary Sciences, Catholic University of the Sacred Heart, 00168 Rome, Italy; 2 Department of Cardiovascular Sciences, Fondazione Policlinico Universitario Agostino Gemelli IRCCS, 00168 Rome, Italy; 3 Department of Cardiology, Zurich University Hospital, 8091 Zurich, Switzerland; 4 Department of Translational Medical Sciences, University of Campania “Luigi Vanvitelli”, 80133 Naples, Italy; 5 Division of Cardiology, A.O.R.N. “Sant’Anna e San Sebastiano”, 81100 Caserta, Italy

**Keywords:** MINOCA, myocardial bridge, pathogenesis, diagnosis, management

## Abstract

Myocardial bridging (MB) is the most frequent congenital coronary anomaly characterized by a segment of an epicardial coronary artery that passes through the myocardium. MB is an important cause of myocardial ischemia and is also emerging as a possible cause of myocardial infarction with non-obstructed coronary arteries (MINOCA). There are multiple mechanisms underlying MINOCA in patients with MB (i.e., MB-mediated increased risk of epicardial or microvascular coronary spasm, atherosclerotic plaque disruption and spontaneous coronary artery dissection). The identification of the exact pathogenetic mechanism is crucial in order to establish a patient-tailored therapy. This review provides the most up-to-date evidence regarding the pathophysiology of MINOCA in patients with MB. Moreover, it focuses on the available diagnostic tools that could be implemented at the time of coronary angiography to achieve a pathophysiologic diagnosis. Finally, it focuses on the therapeutic implications associated with the different pathogenetic mechanisms of MINOCA in patients with MB.

## 1. Introduction

Myocardial infarction with non-obstructed coronary arteries (MINOCA) is defined as the clinical evidence of acute myocardial infarction (MI) with normal or near-normal coronary arteries at coronary angiography (i.e., <50% diameter stenosis in any major epicardial vessel) after excluding systemic non-coronary causes of chest pain and troponin elevation, such as severe anaemia or pulmonary embolism [1,2]. MINOCA may account for up to 10% of patients presenting with acute MI, and, although it was initially considered a benign condition, accumulating evidence has demonstrated that MINOCA patients have worse cardiovascular (CV) outcomes compared with the general population [3,4,5]. Indeed, MINOCA patients have a 1-year mortality and a rehospitalization rate only slightly lower than that of those with acute MI due to obstructive coronary artery disease (CAD) [6]. Furthermore, up to 25% of MINOCA patients might experience angina in the 12 months following the acute event, which has a significant impact on patients’ quality of life and healthcare-related costs [7].

Myocardial bridging (MB) is a congenital coronary anomaly, in which a segment of an epicardial coronary artery, most frequently the left anterior descending artery, deviates its canonical epicardial course by passing through the myocardium [8]. The reported prevalence of MB varies widely according to the different diagnostic tools, ranging from 2–6% at coronary angiography to 19–22% at coronary computed tomography angiography (CCTA) [9,10,11]. Notably, in patients presenting with MINOCA, the prevalence of MB may be as high as 20–40% [12,13,14]. Recent evidence has demonstrated that MB, alone or in combination with other superimposed pathogenetic mechanisms, is a frequent yet commonly overlooked cause of MINOCA [13]. Moreover, the identification of MB and of the pathogenetic mechanism underlying MINOCA may have important therapeutic implications [14].

The purpose of this review is to provide an overview of the most up-to-date evidence regarding the pathophysiology of MINOCA associated with MB. It focuses on the available diagnostic tools that could be implemented during coronary angiography to achieve a pathophysiologic diagnosis. Finally, it focuses on the therapeutic implications associated with the different pathogenetic mechanisms of MINOCA associated with MB.

## 2. Pathophysiology of MB and Its Association with MINOCA

The main feature associated with MB is a dynamic compression of the tunnelled artery during the systole. As coronary blood flow occurs primarily during diastole, only a small percentage (≃15%) of coronary blood flow should theoretically be compromised by the presence of a bridge; accordingly, MB has been long considered a benign condition. However, recent evidence has demonstrated that MB could lead to clinically evident myocardial ischemia through different mechanisms, including the interplay between a high sympathetic tone and a delayed early diastolic coronary artery relaxation [15], the occurrence of a Venturi-like effect along the bridged artery [16], an MB-mediated increased risk of epicardial or microvascular coronary spasm [13,17], atherosclerotic plaque disruption (i.e., plaque rupture or plaque erosion) [18], spontaneous coronary artery dissection (SCAD) [19] or their combination. Each of these mechanisms associated with MB may potentially be the underlying aetiology of MINOCA.

The presence of MB is associated with a delayed early diastolic coronary artery relaxation within the intramyocardial segment and a hindered rapid early diastolic hyperaemia, leading to a reduced coronary blood flow. This mechanism may be particularly relevant in the sub-endocardium, as it is more susceptible to ischemia, and it may be accentuated by an increased sympathetic tone [20,21]. Indeed, the latter increases the heart rate, decreases the diastolic perfusion time, increases the strength of contraction across the MB and delays the relaxation beyond systole into the early diastolic phase, thus impairing coronary blood flow. Moreover, the presence of MB is associated with the occurrence of a Venturi-like effect at the ostium of the side branches, in particular the septal branches, also known as “branch steal”. Indeed, flowing through the tunnelled artery in the end systole/early diastole, the blood passes through a constricted segment leading to an increase in fluid velocity and a decrease in the perfusion pressure at the ostium of the side branches [16]. The key aspects for the risk of myocardial ischemia associated with MB are the depth and the length of the tunnelled segment. Indeed, the depth of MB (<2 mm superficial, ≥2 mm deep, ≥5 very deep) is one of the main determinants of the degree of systolic compression and course of the affected artery. The length of MB is related not only to the amount of affected arteries but also to the number of side branches affected [8].

Of importance is the fact that, as demonstrated by a recent study from our group enrolling patients with myocardial ischemia and non-obstructive coronary arteries undergoing intracoronary provocative testing with acetylcholine (ACh), the prevalence of epicardial or microvascular coronary vasospasm is high among patients with MB (up to 79%, mainly epicardial spasm rather than microvascular spasm). Moreover, coronary spasm coexists more frequently in patients with MINOCA than in stable patients with no obstructive CAD (21.3% vs. 7%, *p* = 0.002). Of note is the fact that the presence of MB was a predictor of MINOCA only in patients with a positive ACh test, and patients with MB and a positive ACh test had a lower major adverse cardiac-event-free survival, representing the group with the worst prognosis [14]. Another study by Nam et al. demonstrated that severe MB (defined as bridge narrowing >90%) was significantly associated with a high risk of coronary spasm, and patients with both conditions were more likely to have recurrent angina compared with MB patients without spasm [17].

Epicardial coronary vasospasm is defined as the vasoconstriction of an epicardial coronary artery caused by vascular smooth muscle hyper-reactivity due to increased sympathetic stimulation, local or systemic inflammation or environmental factors (i.e., smoking, alcohol consumption, environmental exposures) [22]. Microvascular spasm results from an increased constriction of coronary microvessels due to a reduced production and/or enhanced degradation of nitric oxide and other endothelial-derived relaxation factors [23,24]. Mechanistically, the compression–relaxation effect of MB on the coronary arteries may result in injury to the intima and endothelium, leading to impaired endothelium-dependent vasodilatation and enhanced local vascular reactivity to systemic vasoconstrictor stimuli [25]. Moreover, a reduced expression of endothelial nitric oxide synthase has been demonstrated in the MB segment compared to the proximal and distal segments [26]. These alterations also involve coronary microcirculation, as the presence of MB has been associated with alterations in vasoactive agents such as nitric oxide synthase and endothelin-1 and local variations in shear stress [27]. The anatomical features of MB, such as the length and percentage of systolic compression, may further promote the occurrence of coronary vasospasm [28,29]. 

Importantly, the presence of MB might favour the progression and destabilization of atherosclerotic plaques in the segment proximal to the tunnelled one [18]. Plaque rupture is caused by a fibrous cap discontinuity, which exposes the plaque core to the coronary lumen, leading to the activation of a pro-thrombotic cascade and a consequent thrombus formation [30,31]. Plaque erosion is due to the apoptosis of endothelial cells leading to thrombus formation without fibrous cap discontinuity [31,32]. Both plaque rupture and plaque erosion may not determine an angiographically evident flow-limiting stenosis and present as MINOCA through different mechanisms, including a transient thrombosis with spontaneous thrombolysis, distal embolization, superimposed vasospasm or a combination of these processes [33]. In patients with MB, the longstanding compression–relaxation effect of the intramural segment can cause changes in the wall shear stress, leading to the formation of an area of reduced wall shear stress proximal to the MB and the release of inflammatory mediators and endothelial vasoactive agents [25]. Conversely, the wall shear stress is increased along the tunnelled artery, thus sparing this segment from atherosclerosis. Another mechanism that could promote atherosclerotic plaque formation and progression in the segment proximal to the MB is the abnormal blood flow profiles in this area caused by the collision of anterograde coronary flow with the retrograde flow due to the compression of the MB during the systole. Such alterations can determine endothelial injury, thus promoting plaque formation and disruption [34].

The presence of MB might increase the risk of SCAD, a frequently overlooked cause of MINOCA, especially in young women. SCAD is caused by the separation of the layers of an epicardial coronary artery wall by intramural haemorrhage, with or without an intimal tear, not associated with atherosclerosis, iatrogenic injury or trauma, leading to the formation of a false lumen wherein the axial propagation of blood flow may result in a narrowing of the true lumen. The exact pathophysiology of SCAD is complex and likely involves multiple predisposing and precipitating factors, including genetic predisposition, hormonal influences, vascular and systemic inflammation, intense exercise, emotional stress and drug abuse [35,36]. Different case reports showed that SCAD might occur in patients with MB even in the absence of other predisposing or precipitating factors and tends to localize within the intramyocardial segment or distal to the MB [37,38]. Several mechanisms may be involved, including the occurrence of coronary vasospasm in the bridged segment that could increase its susceptibility to dissection or the presence of MB-induced endothelial dysfunction [19,39]. Moreover, the flow disturbance caused by the pressure gradient between the bridged and non-bridged segment can lead to a state of chronic coronary pressure overload, which may cause intimal trauma, which eventually leads to intimal tearing and coronary dissection [40].

Finally, previous studies have reported that MINOCA might be associated with a low but still not neglectable rate of complications during the acute phase, mainly represented by intraventricular septal rupture, free wall rupture with pericardial effusion or cardiac tamponade and ventricular arrhythmias [41]. A case of ventricular septal rupture caused by MINOCA has recently been reported, in which a septal branch steal phenomenon might have contributed to its occurrence [42]. However, to date, there are no data reporting the risk of mechanical complications in MB-related MINOCA, likely because this association is still frequently underdiagnosed in clinical practice.

## 3. Invasive Diagnostic Approach to Patients with MINOCA and MB

**Figure 1:**
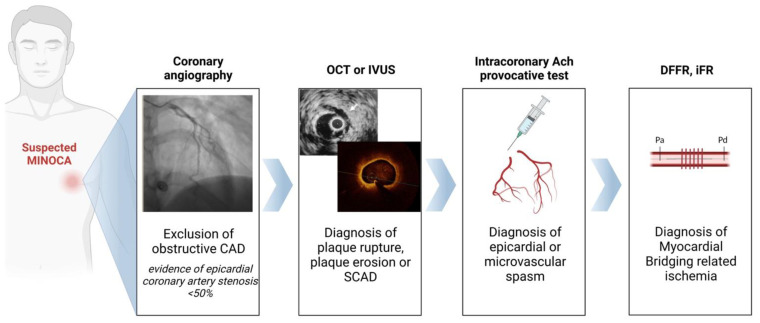
Invasive diagnostic algorithm in patients with MINOCA associated with myocardial bridging. Abbreviations: CAD: coronary artery disease; DFFR: diastolic fractional flow reserve; iFR: instantaneous wave-free ratio; IVUS: intravascular ultrasound; MINOCA: myocardial infarction with non-obstructed coronary arteries; OCT: optical coherence tomography; SCAD: spontaneous coro-nary artery dissection.

Coronary angiography is the cornerstone for the diagnosis of MINOCA by excluding the presence of stenosis of more than 50% in any major epicardial coronary artery. The use of intracoronary vasodilators during diagnostic coronary angiography can increase the systolic “milking” of the tunnelled artery induced by the systolic compression of the intramural artery and facilitate the detection of MB [43,44]. A significant “milking effect” is present when there is a visual >70% reduction in the minimal luminal diameter during systole and a persistent >35% reduction in the minimal luminal diameter during mid-to-late diastole. Once the diagnosis of MINOCA and MB has been established or suspected, the use of advanced diagnostic techniques might be performed at the time of coronary angiography, aiming at confirming the presence of MB and identifying the presence of one of the above-described mechanisms of MINOCA associated with MB (Figure 1).

Intracoronary imaging techniques (i.e., intravascular ultrasound (IVUS) or optical coherence tomography (OCT)) are extremely helpful in this setting. IVUS is an ultrasound-based technology that allows the detection of the systolic compression of the intramyocardial segment due to MB and of an echo lucent area between the intramyocardial tract of the coronary artery and the epicardium known as a “half-moon” sign, which represents the muscle band overlying the intramyocardial segment [45]. Moreover, IVUS may allow the detection of atherosclerotic plaque rupture or SCAD. OCT is a light-based technique that provides high in vivo resolution (~10 µm) images of the coronary artery that are approximately ten times greater than those of IVUS [46]. Therefore, OCT has a higher diagnostic accuracy for the presence of plaque rupture, plaque erosion or SCAD, but it has a lower diagnostic accuracy for the detection of MB, mainly because of its limited penetration and rapid OCT pullback and image acquisition (20 mm/s vs. 0.5 mm/s in IVUS). However, the presence of a heterogeneous fusiform band with sharp borders and a low-/intermediate-intensity signal surrounding the vessel adventitia has been described in the presence of MB (similar to the “half-moon” sign in IVUS), but further research is needed for its validation [47,48]. IVUS and OCT show plaque rupture as a discontinuity of the fibrous cap overlying a lipid-rich core. A ruptured fibrous cap is associated with a vessel wall cavity without an IVUS or OCT signal [49,50]. A thrombus often overlies the ruptured segment but may be absent in case of an old plaque rupture or with a recent rupture treated with antithrombotic therapies. Conversely, in the case of plaque erosion, OCT shows the absence of fibrous cap disruption in a lesion that is frequently composed of fibrous tissue with an overlying luminal white thrombus [49]. IVUS and OCT can also be used to facilitate the diagnosis of SCAD, showing the intramural haematoma with or without the intimal flap [49].

Intracoronary provocative tests using pharmacologic vasoactive agents can elicit a vasoconstrictive response at both the epicardial and microvascular level and, therefore, are fundamental for the diagnosis of coronary vasomotor disorders. ACh is usually preferred over ergonovine as a provocative agent due to its lower rate of complications. Moreover, performing an ACh-provocative test in MINOCA is safe with a relatively low risk of transient complications, which are mainly represented by transient bradyarrhythmia and supraventricular tachycardia [51,52,53]. Given that ACh, by binding muscarinic 3 receptors, stimulates nitric oxide release from the endothelium to vasodilate and directly induce vascular smooth muscle cells (VSMCs) contractility, the net effect of intracoronary ACh administration is either vasodilatation (healthy endothelium) or coronary spasm (endothelial dysfunction or increased VSMCs reactivity) [54,55,56]. This test is considered positive for epicardial coronary spasm in the presence of: (1) focal or diffuse epicardial coronary diameter reduction ≥90% in comparison to the relaxed state following intracoronary nitroglycerine administration given to relieve the spasm; (2) the reproduction of the patient’s symptoms; and (3) ischemic ECG shifts [57]. On the other hand, microvascular spasm is diagnosed when typical ischemic ST-segment changes and angina develop in the absence of epicardial coronary constriction (<90% diameter reduction) [58].

If other aetiologies have been excluded, the dynamic stenosis caused by MB may be responsible for myocardial ischemia and troponin release in particular circumstances (hypertensive crisis, tachyarrhythmias, etc.). To assess the hemodynamic significance of MB-induced stenosis, the use of several techniques has been suggested. Fractional flow reserve (FFR) is a pressure-wire-based index that is routinely used to assess the hemodynamic relevance of intermediate coronary stenosis. FFR is obtained through the calculation of the ratio of mean blood pressure distal to coronary stenosis to the mean aortic pressure during maximal hyperaemia achieved by the intravenous administration of adenosine (cut-off value of ≤0.80) [59]. However, FFR may not be accurate for the assessment of MB as it is based on the assumption that the difference between the mean and diastolic pressure gradient values across the lesion is neglectable at maximal hyperaemia. Indeed, the presence of MB reduces systolic pressure gradients because of distal pressure overshooting during myocardial contraction, with the consequent overestimation of the mean pressure and, consequently, the underestimation of the hemodynamic impact of MB [60]. Partially overcoming these limitations, diastolic FFR (dFFR), which is only calculated from diastolic pressures and is obtained during the maximal hyperaemia induced by intracoronary adenosine, has been demonstrated to be more sensitive than FFR for the functional assessment of MB. Escaned et al. found that, among twelve patients with symptomatic MB, dFFR identified a hemodynamic relevance in five patients, whereas mean FFR did so in only one patient, and the discrepancy between mean FFR and dFFR increased with dobutamine challenge [61]. Indeed, as the dynamic obstruction caused by MB is deeply influenced by the degree of extravascular compression and intramyocardial tension that is increased during exercise or stress, its evaluation at rest may underestimate the hemodynamic relevance. For this reason, inotropic stimulation with dobutamine may enhance the diagnostic sensitivity of dFFR, and a cut-off value of ≤0.76 for dFFR has been proposed during dobutamine provocation to identify stress-induced myocardial ischemia in MB patients [61]. However, dFFR is still not routinely performed because it is time-consuming, mainly due to the need for hyperaemic stimulus with adenosine, inotropic stimulation with dobutamine and data post-processing for its calculation [62]. Additionally, non-hyperaemic pressure indices not requiring the administration of adenosine have been developed, including the instantaneous wave-free ratio (iFR), a diastolic-specific flow index. Tarantini et al., in a prospective study including 20 patients with angina and an abnormal non-invasive test suggestive of ischemia, demonstrated that iFR was associated with a higher proportion of positive obstructive bridges compared to FFR [63]. Of note is the fact that iFR has only been formally validated in patients without MB with a cut-off value considered to be <0.89, while the measurement iFR in patients with MB in both rest and stress states (i.e., during inotropic infusion) requires further validation studies [8].

Finally, the non-invasive functional evaluation at rest of MB with computed tomography (CT)-derived FFR has been investigated (cut-off value 0.75, grey zone 0.75–0.80) [64]. However, further studies are needed for its validation, and, moreover, this approach is affected by the same limitations that apply to invasive FFR, especially in MINOCA patients [8]. Indeed, during the acute phase, microvascular and endothelial dysfunction may be present, raising concerns regarding the achievement of maximal hyperaemia and, consequently, the reliability of FFR that can lead to the underestimation of the hemodynamic significance of MB [56]. Similarly, cardiac magnetic resonance imaging could be used to assess for segmental myocardial perfusion defects and allow the physiological assessment of the functional effects of MB, but its routine use is limited by technical challenges and a lack of spatial resolution [65]. In the context of MINOCA, cardiac magnetic resonance, thanks to its high accuracy in discriminating between ischaemic or non-ischaemic aetiologies, may help in the differential diagnosis between myocardial infarction, inflammatory cardiac diseases and Takotsubo syndrome [50].

## 4. Therapeutic Implications

The management of MINOCA still has a limited number of evidence-based studies, as only a few registries have addressed this issue, and there is a strong need for randomized controlled trials in this setting [66,67]. Adding complexity, specific therapeutic considerations are likely needed in patients presenting with MINOCA in which a specific pathogenetic mechanism associated with MB is diagnosed (Table 1). However, there is a lack of evidence supporting any specific intervention as well as reporting the incremental prognosis of MINOCA associated with a MB. Accordingly, there is a strong need for properly designed randomized clinical trials as well as for data from observational studies or registry to support recommendations in future clinical consensus or guidelines in these patients.

### 4.1. Pharmacological Treatment

β-blockers are considered the first-line therapy in MB because of their negative chronotropic and inotropic effects and the fact that they decrease the heart rate and increase the diastolic filling time, thus allowing the decompression of the intramyocardial segment [68]. Nebivolol could be the best choice due to its highly selective beta 1 blockade and the possible beneficial effects on endothelial function via the stimulation of beta 3 receptors [69]. However, β-blockers may worsen the occurrence of epicardial or microvascular spasm favouring coronary vasoconstriction by unmasking α-adrenoreceptors in the coronary circulation. Therefore, in patients with MINOCA and MB in whom an ACh test is positive for epicardial or microvascular spasm, β-blockers could be contraindicated because they can worsen the extent of spasm, while calcium channel blockers (CCBs) should be used for their vasodilatory effect [25]. Ivabradine may play a role as a second-line agent, particularly in patients unable to tolerate β-blockers/CCBs or those who do not achieve an adequately controlled heart rate on maximally tolerated treatment because of its ability to lower the heart rate [70].

### 4.2. Percutaneous Coronary Intervention (PCI)

PCI can be performed in patients with MINOCA caused by atherosclerotic plaque rupture associated with MB [71]. However, PCI in the context of MB may be technically challenging and portend a higher risk of short- and long-term complications [72]. On the other hand, plaque erosion might be stabilized with DAPT without stenting, according to recent evidence [73,74]. In addition, these patients should be treated with high-dose statins [75].

In patients with MINOCA and SCAD associated with MB, PCI may contribute to the propagation of the dissection and mural hematoma and, therefore, is not routinely performed. However, PCI should be considered if there is evidence of ongoing ischemia, hemodynamic instability, ventricular arrhythmias or left main dissection [76]. At the same time, the pharmacotherapy of SCAD remains controversial, with no clinical trials to date supporting specific therapeutic recommendations. However, pharmacological therapy is usually based on acetylsalicylic acid and β-blockers, while the role of DAPT in patients without PCI is still matter of debate, as the results of a European registry suggest that DAPT, compared with single antiplatelet therapy, is associated with a higher rate of adverse cardiovascular events at 1 year [77].

Finally, if all the other causes have been excluded and MB-related ischemia is identified as the underlying mechanism responsible for the MINOCA, PCI of the MB can be considered to protect the intramyocardial segment from systolic compression, especially in patients with refractory anginal symptoms despite optimal medical therapy. PCI has been associated with both hemodynamic and symptom improvements in MB [78]. The use of high-radial-strength second-generation drug-eluting stents combined with accurate stent sizing using intravascular imaging may potentially improve outcomes in this setting by reducing the risk of stent fracture, stent thrombosis, perforation during stent deployment and in-stent restenosis [71]. Specific factors favouring PCI in MB include a shorter lesion length (<25 mm) and a more superficial depth (<2 mm) of the tunnelled segment.

### 4.3. Surgical Treatment

In the case of deeper/longer MBs identified as being responsible for MINOCA, especially if requiring more than a single stent, surgical options should be considered, including coronary artery bypass grafting (CABG) or supra-arterial myotomy (also known as “unroofing”) [79]. CABG can be performed on or off a cardiopulmonary bypass, and either arterial or saphenous vein grafts can be used. MB of the left anterior descending artery (LAD) is commonly bypassed by using the left internal mammary artery. The main concern of CABG in MB is graft failure, likely due to competitive flow [80]. Recently, a novel technique in which a free graft of the left internal mammary artery is anastomosed to the LAD proximal and distal to the MB has been described, but further studies are needed [81]. Supra-arterial myotomy consists of the dissection of the tunnelled artery from the overlying myocardium, with the potential benefit of restoring the physiological anatomy. However, a high incidence of recurrent angina has been reported, likely due to the persistence of endothelial dysfunction [82]. Therefore, the benefits of myotomy could be higher in the paediatric population where the alterations associated with MB have had less time to develop. Evidence comparing CABG to myotomy are still limited, and, therefore, clear recommendations could not be provided. CABG has previously been favoured over myotomy in cases of extensive (>25 mm) or deep (>5 mm) MB or when the coronary segment fails to decompress completely in diastole. A recent observational study of 54 patients with symptomatic MB of the LAD undergoing surgery, CABG was associated with a significantly higher rate of major adverse cardiovascular events (MACEs) at a median follow-up of 26 months (7.4% for myotomy vs. 40.9% for CABG, *p* = 0.007), and surgical strategy (CABG vs. myotomy) was an independent risk factor for MACEs. Furthermore, on follow-up CCTA, nine of the twenty-three patients who underwent CABG experienced graft failure, but all ten patients who had >50% proximal stenosis on preoperative angiography were found to have patent grafts on follow-up. This observation likely suggests the role of competitive flow as a major factor contributing to CABG patency in patients with MB, and, therefore, CABG may be recommended for treating symptomatic MB with concomitant proximal coronary stenosis [83]. Furthermore, if CABG is planned as the upfront treatment of MB, saphenous venous grafting should be considered [84].

## 5. Conclusions and Future Directions

MB and MINOCA are two very common clinical entities, where the former may lead to the latter through the above-described pathophysiological mechanisms. The accurate diagnosis of the underlying mechanisms through multiple investigations, such as OCT, IVUS and pressure wire methods, can lead to a pathophysiology-driven approach with targeted therapies according to the specific mechanism of MINOCA associated with MB. There is a strong need for evidence coming from properly designed randomized clinical trials as well as observational studies to clarify whether such a personalized approach could improve patients’ outcomes in this setting and support the formulation of clear recommendations in future clinical guidelines.

## Figures and Tables

**Table 1:** Therapeutic implications according to the underlying pathogenetic mechanism of MINOCA associated with a MB.

Pathogenetic Mechanism of MINOCAAssociated with a MB	Therapeutic Implications
Plaque rupture	- PCI with stent implantation. - DAPT for 12 months followed by SAPT with acetylsalicylic acid. - High-dose statins.
Plaque erosion	- A conservative strategy (PCI without stent implantation) and DAPT for 12 months followed by SAPT may be feasible and potentially translate in a reduction in both early and late stent-related complications. - High-dose statins.
SCAD	- PCI should be considered if there is evidence of ongoing ischemia, hemodynamic instability, ventricular arrhythmias or left main dissection. - SAPT with acetylsalicylic acid and β-blockers.
Epicardial coronary spasm	- β-blockers contraindicated
Microvascular coronary spasm	- Dihydropyridine CCBs - Ivabradine
MB-related ischaemia	- β-blockers (first line) or dihydropyridine CCBs if contraindications to β-blockers (e.g., bronchospasm). - Ivabradine (second line). - PCI or CABG of the MB segment (third line).

Abbreviations: MINOCA: myocardial infarction with non-obstructed coronary arteries; MB: myocardial bridge; PCI: percutaneous coronary intervention; DAPT: dual antiplatelet therapy; SAPT: single antiplatelet therapy; SCAD: spontaneous coronary artery dissection; CCBs: calcium channel blockers; CABG: coronary artery bypass grafting.

## Data Availability

Not applicable.
